# Prediction of formation force during single-point incremental sheet metal forming using artificial intelligence techniques

**DOI:** 10.1371/journal.pone.0221341

**Published:** 2019-08-22

**Authors:** Ali Alsamhan, Adham E. Ragab, Abdulmajeed Dabwan, Mustafa M. Nasr, Lotfi Hidri

**Affiliations:** King Saud University, Industrial Engineering Department, King Saud University, Riyadh, Saudi Arabia; Newcastle University, UNITED KINGDOM

## Abstract

Single-point incremental forming (SPIF) is a technology that allows incremental manufacturing of complex parts from a flat sheet using simple tools; further, this technology is flexible and economical. Measuring the forming force using this technology helps in preventing failures, determining the optimal processes, and implementing on-line control. In this paper, an experimental study using SPIF is described. This study focuses on the influence of four different process parameters, namely, step size, tool diameter, sheet thickness, and feed rate, on the maximum forming force. For an efficient force predictive model based on an adaptive neuro-fuzzy inference system (ANFIS), an artificial neural network (ANN) and a regressions model were applied. The predicted forces exhibited relatively good agreement with the experimental results. The results indicate that the performance of the ANFIS model realizes the full potential of the ANN model.

## Introduction

Incremental sheet forming (ISF) is a flexible manufacturing technology that does not require special dies and uses a single tool to produce a variety of regular and multifaceted shapes. Further, it is economical when employed in the manufacturing of complex parts using simple tools as compared with other conventional sheet metal forming technologies (e.g., extrusion, hydroforming [1], and deep drawing [2]) because it does not require expensive dies or punches. In addition, it is used as a simple tool to incrementally develop the desired parts from sheets. However, it is time-consuming and thus not useful for mass production.

The ISF technique can be separated into two classes: two-point incremental forming (TPIF), which requires a partial die as a support for the sheet during the process [3], and single-point incremental forming (SPIF), which does not require any specific die [4]. Currently, focus is on SPIF, in which a small hemispherical tool is used to mold the sheet into the desired shapes; the tool is driven using a computer numerical control (CNC) machine along a predefined toolpath generated through computer-aided manufacturing software. The peripheral of the sheet is clamped using a fixture. Through this technique, complex parts can be manufactured in small batches and prototypes can be economically obtained.

ISF is used in many applications, such as the manufacturing of automotive parts [5] and the standardization of void nucleation models for automotive aluminum sheets [6]. Furthermore, it can be used to produce parts such as a palate or knee implants [7] or an ankle prosthesis [8] [9]. The forming force in SPIF is essential while utilizing machines adapted for processes such as robots and milling centers [3]. It helps determine the optimal process parameters and equipment suitable for sheet forming [10]. The forming force has characteristics that are essential in predicting the power of a machine; in addition, it helps with the design of tools and improves the understanding of the deformation mechanics of several processes [11]. Iseki [12] was among the first few researchers to determine the forming forces for a pyramid based on a plane–strain deformation using a simple approximated deformation analysis. Later, Jeswiet et al. [13] measured the force magnitudes of SPIF and TPIF pyramids and truncated cones. Filice et al. [14] worked on a force analysis and categorized the force trends of a tangential force into three categories, namely, monotonically reducing, polynomial, and steady-state force trends. Dabwan [15] showed that the sheet thickness is the main factor in estimating the forming force, followed by the tool diameter and step size. The feed rate has proven to be insignificant in estimating the forming force. Duflou et al. [16] found that the forming forces increase with the sheet thickness, wall angle, and step size. Kumar and Gulati [17] investigated and optimized the effects of input factors on the forming forces using the Taguchi approach and analysis of variance. They showed that the force trend after the peak values depends on the instant input factors, which can be categorized into sets of parameters such as safe, severe, and crucial. Bagudanch et al. [18] concluded that the forming force is influenced by the bending condition. They also found that the forming force decreases as the spindle speed increases. Arfa et al. [19] and Henrard et al. [20] used a finite element analysis to predict the SPIF forces with satisfactory precision. Ingarao et al. [21] calculated and estimated the energy consumption for the SPIF process based on the recorded force data. Petek et al. [22] studied and localized the fracture by analyzing the response force using a skewness function. Fiorentino [23] presented another failure criterion according to the force detected during the forming process. Moreover, Ambrogio et al. [24] proposed that the incremental increase in force required to reach its maximum value can be effectually used as a predecessor to failure in SPIF.

Therefore, it is essential to model and quantify the relationship between the forming force and the input process parameter affecting its value. Further, empirical models developed using traditional methods may not describe the nonlinear complex relationship between the input and output variables. Fuzzy logic (FL), an artificial neural network (ANN), and a genetic algorithm are unconventional methods used to develop models for a nonlinear complex system. An adaptive neuro-fuzzy inference system (ANFIS) can be used in numerous fields such as manufacturing technologies, machining, and economic systems [25]. ANFIS is a type of ANN developed based on a Takagi–Sugeno FIS. This approach was developed during the 1990s. ANFIS is a combination of neural networks and FL principles, and can capture the benefits of both in a single framework [26]. The inference system corresponding to the set of fuzzy IF-THEN rules can approximate nonlinear functions [27]. Therefore, ANFIS is considered a comprehensive estimator.

An investigation into the forming forces in SPIF is particularly important for selecting the appropriate hardware and optimizing the process parameters to assure the precision of a process. The efficient prediction of the forming forces is desirable in order to monitor the forming process, prevent failures, and implement on-line process control. The characterization of the forming forces is essential in order to estimate the needed power of the machine. The expected forming force has consequences regarding the design of the tooling and fixtures, as well as on the selected machine. There has recently been an increasing interest in the development of models that can help investigate the effects of input variables on the performance outputs using artificial intelligence methods as an alternative to traditional approaches [28]–[31]. This paper proposes an intelligent process model, founded on the concept of data mining, for predicting the forming forces in SPIF. Several researchers have addressed the limitations of this process, resulting in low-quality profile products. The predictive model for the forming forces described in this paper is based on an adaptive-neuro fuzzy inference system (ANFIS) and an artificial neural network (ANN), which have not been considered in previous studies to the best of our knowledge. An accurate model used to predict the forming forces in SPIF is essential in order to control the process quality.

The rest of this paper is organized as follows: The experiments are presented in Section 2. The ANFIS, ANN, and regression models are presented in Sections 3 and 4. The results and discussions are detailed in Section 5. Finally, the conclusions are presented in Section 6.

## Experiments

A vertical CNC milling machine, a specially designed fixture, forming tools, and a piezoelectric dynamometer were used to conduct the experiments. The sheet material selected for this study was a commercial aluminum alloy, AA1050-H14, which is a popular grade of aluminum for general sheet metal work owing to its excellent corrosion resistance, high ductility, and highly reflective finish. Further, the material composition, extracted using a SPECTRO machine, is presented in Table 1. Tensile tests were conducted on the specimens using a Zwick/Roell universal testing machine, the results of which are presented in Table 2. The sheet was clamped using the designed fixture in a working area of 200 mm × 200 mm. The tool used during this process was cylindrical with a hemispherical head. In this study, the tool motion was controlled numerically. Therefore, the required part was designed using SOLIDWORK software, and the design was then transferred to MASTERCAM software to generate the toolpath. The numerical control (NC) codes were obtained from the generated toolpath and transferred to the CNC machine. For the accurate formation of parts, it is important to select the best toolpath, which in this case is a spiral toolpath. A truncated conical geometry was built with a base diameter of 100 mm and a height of 50 mm. Important parameters considered for the incremental sheet metal forming are tool diameter, sheet thickness, feed rate, and step size, the values of which are listed in Table 3.

**Table 1:** Chemical composition of AA1050-H14 sheets used in this study.

Sample	Al %	Fe %	Si %	Ti %	Other
1	99.5	0.368	0.0480	0.0216	0.0624
2	99.5	0.360	0.0496	0.0205	0.0007

**Table 2:** Measured mechanical properties of aluminum alloy AA1050-H14.

Material code	Yield Strength σy (MPa)	Ultimate Tensile Strength σUTS (MPa)	Elongation at Break A (mm)	Young Modulus E (MPa)
AA1050-H14	128	117.5	8.45	67648

**Table 3:** Process parameters and their levels.

Input process parameters	Level 1	Level 2
Tool diameter (d)	10 mm	20 mm
Feed rate (f)	500 mm/min	1000 mm/min
Step size (s)	0.5 mm	1 mm
Sheet thickness (t)	1 mm	2 mm

**Fig 1:**
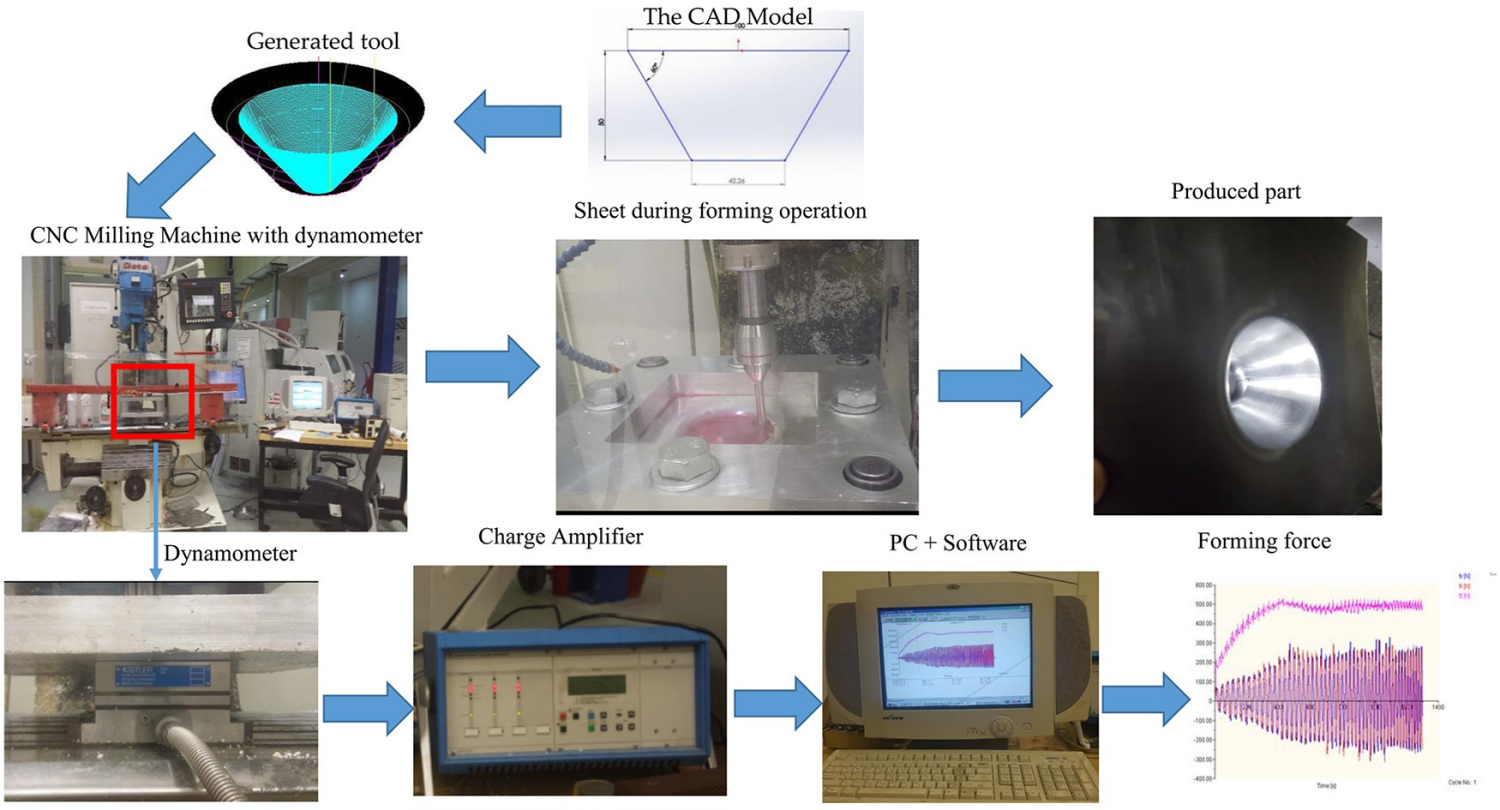
Experimental setup and forming force measurement.

Measuring the forming force during this process is extremely important to prevent failure, determine the optimal process, and implement on-line control. Forming force tests were conducted using a KISTLER 2825A1 with eight freely selectable measuring signal-component force dynamometer controllers, which helped measure the force components in three directions (x, y, and z). In addition, the measuring system included charge amplifiers (a complementary KISTLER 5019B three-channel charge amplifier) and data acquisition cards to record the measured forces on a PC. The sampling rate of the force measurement was 50 Hz. Fig 1 shows the experimental system and procedure used to measure the performance of the forming forces. The workpiece fixture was mounted on top of a piezoelectric load cell. The experimental results for all responses that were used as training and testing data for both the ANN and ANFIS models are listed in Table 4.

**Table 4:** Process parameters used with the corresponding experimental results of forming forces and predicted results.

Input Parameters				Experiment forming force			Predicted by ANFIS			Predicted by Regression			Predicted by ANN		
D	f	S	t	Fx (N)	Fy (N)	Fz (N)	Fx (N)	Fy (N)	Fz (N)	Fx (N)	Fy (N)	Fz (N)	Fx (N)	Fy (N)	Fz (N)
10	500	1	1	318.16	331.69	529.75	313	288	528	305.1563	313.846	393.83	133.6167	682.762	535.5682
20	1000	0.5	2	680.92	705.17	1636.29	681	692	1600	679.9625	718.428	1701.837	325.2577	305.312	1617.228
20	1000	0.5	1	313.98	304.99	525.67	318	305	562	316.9325	347.377	558.8969	177.1499	777.368	570.9501
20	500	0.5	2	779.79	770.42	1637.1	668	779	1600	751.625	709.889	1554.133	177.4208	200.635	1644.425
10	500	0.5	1	200.51	202.94	427.65	199	203	451	180.7838	196.024	484.765	194.3887	286.471	532.8672
10	500	0.5	1	197.09	214.55	474.92	199	203	451	180.7838	196.024	484.765	194.3887	239.491	532.8672
10	500	1	1	307.23	288.38	449.73	313	288	528	305.1563	313.846	393.83	133.6167	777.368	1644.425
20	500	0.5	1	323.41	323.65	520.47	372	249	296	388.595	261.341	411.1931	182.1449	168.643	417.5815
20	500	0.5	2	756.54	788.15	1651.34	668	779	1640	751.625	709.889	1554.133	177.1499	682.762	1617.228
10	1000	0.5	1	187.17	186.83	375.34	187	187	375	163.1713	157.298	359.975	202.9756	124.666	1644.413
20	1000	0.5	2	677.69	678.64	1587.21	681	692	1610	679.9625	718.428	1701.837	180.4025	124.666	319.592
10	1000	0.5	1	186.81	188.07	411.57	187	187	375	163.1713	157.298	359.975	134.2937	239.491	566.8363
20	500	1	2	420.7	447.42	873.51	338	365	770	449.615	466.56	934.7656	198.0989	373.817	584.791
20	500	0.5	1	420.7	173.59	295.76	372	249	296	388.595	261.341	411.1931	177.4208	152.6	509.3007
10	500	1	2	377.02	380.88	724.6	352	381	674	359.5638	295.91	685.505	246.7695	241.052	1618.019
20	1000	1	1	140.51	143.01	219.14	148	146	226	201.9425	106.683	304.7819	182.1449	605.412	363.1248
10	500	0.5	2	222.86	262.58	472.52	223	246	558	235.1913	315.321	565.99	180.4025	203.033	412.5591
10	1000	0.5	2	195.17	236.15	469.88	194	215	492	217.5788	199.098	441.2	173.8994	152.6	1618.019
20	1000	1	2	862.29	868.92	1631.76	619	612	1250	564.9725	594.368	1082.469	139.1313	149.039	570.9501
10	1000	1	2	258.35	270.47	564.92	258	292	572	208.5413	298.957	560.715	198.0989	147.722	363.1248
20	1000	1	2	375.95	355.04	863.46	619	612	1250	564.9725	594.368	1082.469	202.9756	277.943	566.8363
20	1000	0.5	1	321.2	329.46	598.28	318	305	532	316.9325	347.377	558.8969	130.9983	147.722	416.6108
10	500	1	2	327.03	331.91	622.96	352	381	674	359.5638	295.91	685.505	139.1313	152.6	251.8233
10	1000	1	2	257.48	292.22	579.96	258	292	572	208.5413	298.957	560.715	173.7338	124.666	584.791
20	500	1	1	157.15	160.28	239.35	157	159	237	86.585	155.244	157.0781	130.9983	241.052	1644.413
20	1000	1	1	155.08	148.48	234.16	148	146	226	201.9425	106.683	304.7819	189.5676	149.039	509.3007
20	500	1	2	338.47	364.73	665.74	338	365	770	449.615	466.56	934.7656	246.7695	361.18	412.5591
10	1000	0.5	2	192.35	215.09	513.93	194	215	492	217.5788	199.098	441.2	130.5799	305.312	615.1648
10	1000	1	1	112.52	111.6	176.58	113	101	174	154.1338	140.523	269.04	134.2937	373.817	251.8233
20	500	1	1	156.08	157.83	231.07	157	159	237	86.585	155.244	157.0781	130.5799	200.635	412.5591
10	1000	1	1	97	91.32	169.68	113	101	174	154.1338	140.523	269.04	173.7338	203.033	417.5815
10	500	0.5	2	211.49	229.27	558.05	223	246	558	235.1913	315.321	565.99	189.5676	277.943	319.592

## Development of predictive models for forming force

### Adaptive neuro-fuzzy inference system

**Fig 2:**
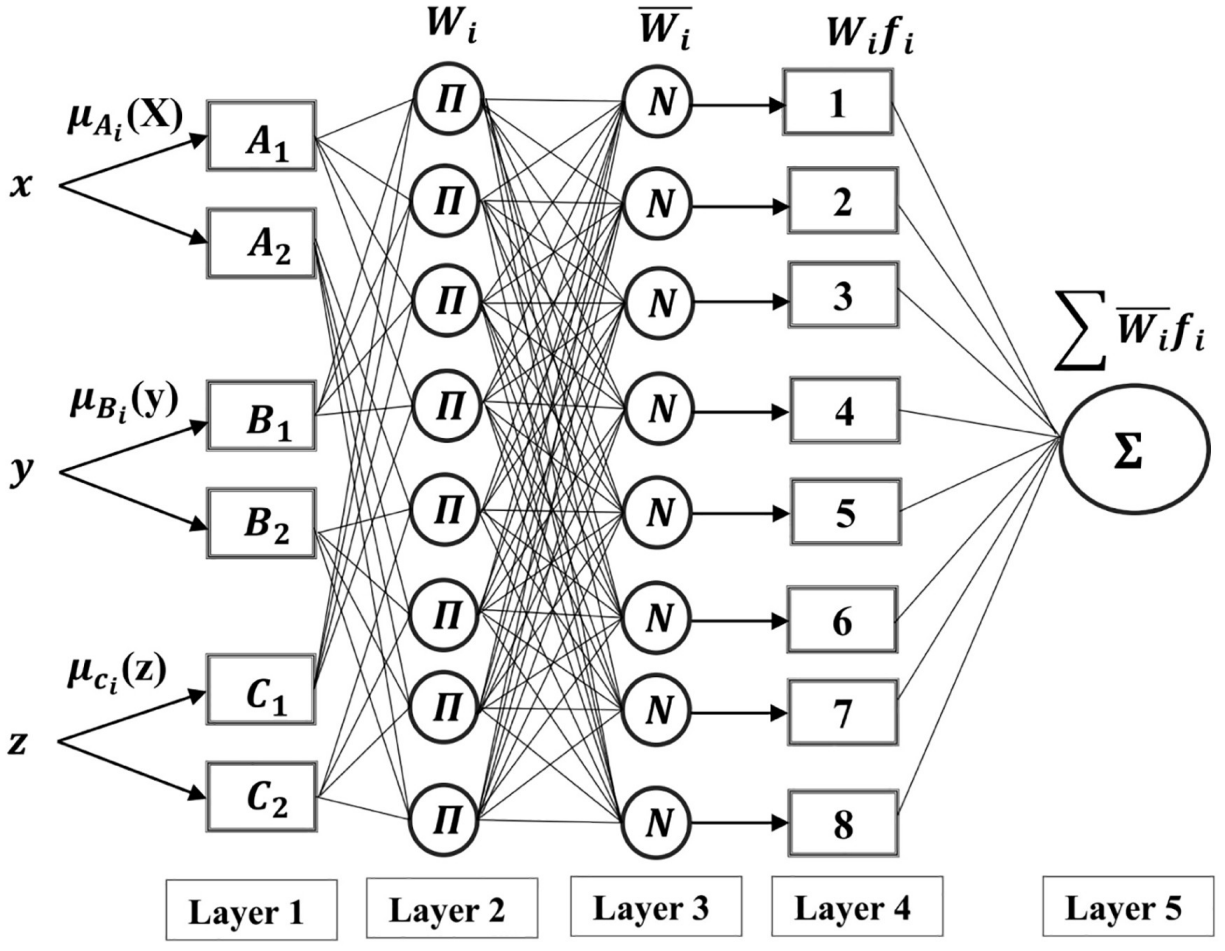
ANFIS architecture with five layers and several nodes [32].

ANFIS is an effective approach to building models of complex nonlinear systems. Here, a hybrid learning process is used to structure an input–output mapping based on human knowledge and training data pairs. The ANFIS is applied in the framework of adaptive networks. It consists of five network layers. Each layer is described by several node functions. The information is moved unidirectionally. A diagram of the ANFIS structure with three inputs and two membership functions for each input and one output is shown in Fig 2. The objective of the current work is to investigate the potential of ANFIS in SPIF.

ANFIS consist of five layers to achieve the following fuzzy inference [32]:

Layer 1: Fuzzy layer
  In this layer, the membership value is calculated using the following equation:
  $$\mu Ai(x)=\frac{1}{1+[{{\left(\frac{x-c_{i}}{a_{i}}\right)}^{2}]}^{b_{i}}},$$ (1)
  where μ A_i_(x) is an appropriate parameterized membership function, and a_i_, b_i_, and c_i_ form a parameter set that changes the forms of the functional movement screen () with a value between 1 and 0.
Layer 2: Multiplies the incoming signals and sends the product out.
  $$i=\mu Ai(x)\;\times \mu \;B_{i}(y)\times \;\mu \;C_{i}(z),\;i=1,2,$$ (2)
  Each node output represents the firing strength of a rule.
Layer 3: Normalizes the firing strengths
  In this layer, the normalized firing strength is computed using the following equation:
  $${\overset{-}{w}}_{i}=\frac{w_{i}}{\sum_{i}w_{i}}\;,\;i=1,2,$$ (3)
  where w_i_ denotes the output of layer i.
Layer 4: Defuzzification
  In this layer, each node i is an adaptive node with a node function.
  $${\overset{-}{w}}_{i}.f_{i}={\overset{-}{w}}_{i}.(p_{i}.x+q_{i}.y+r_{i}.z+s_{i}),$$ (4)
  where p_i_, q_i_, r_i_, and s_i_ make up the consequent parameter set of the node, which are identified during the training process.
Layer 5: Total output layer
  In this layer, all incoming signals are added (summation output). The circle node function is fixed whereas the indicated square function is adaptive. This can be calculated as follows:
  $$Overall\;output=\sum_{i}{\overset{-}{w}}_{i}.f_{i}=\frac{\sum_{i}w_{i}.f_{i}}{\sum_{i}w_{i}}\;,\;i=1,2,$$ (5)

### Neural network model used for prediction

**Fig 3:**
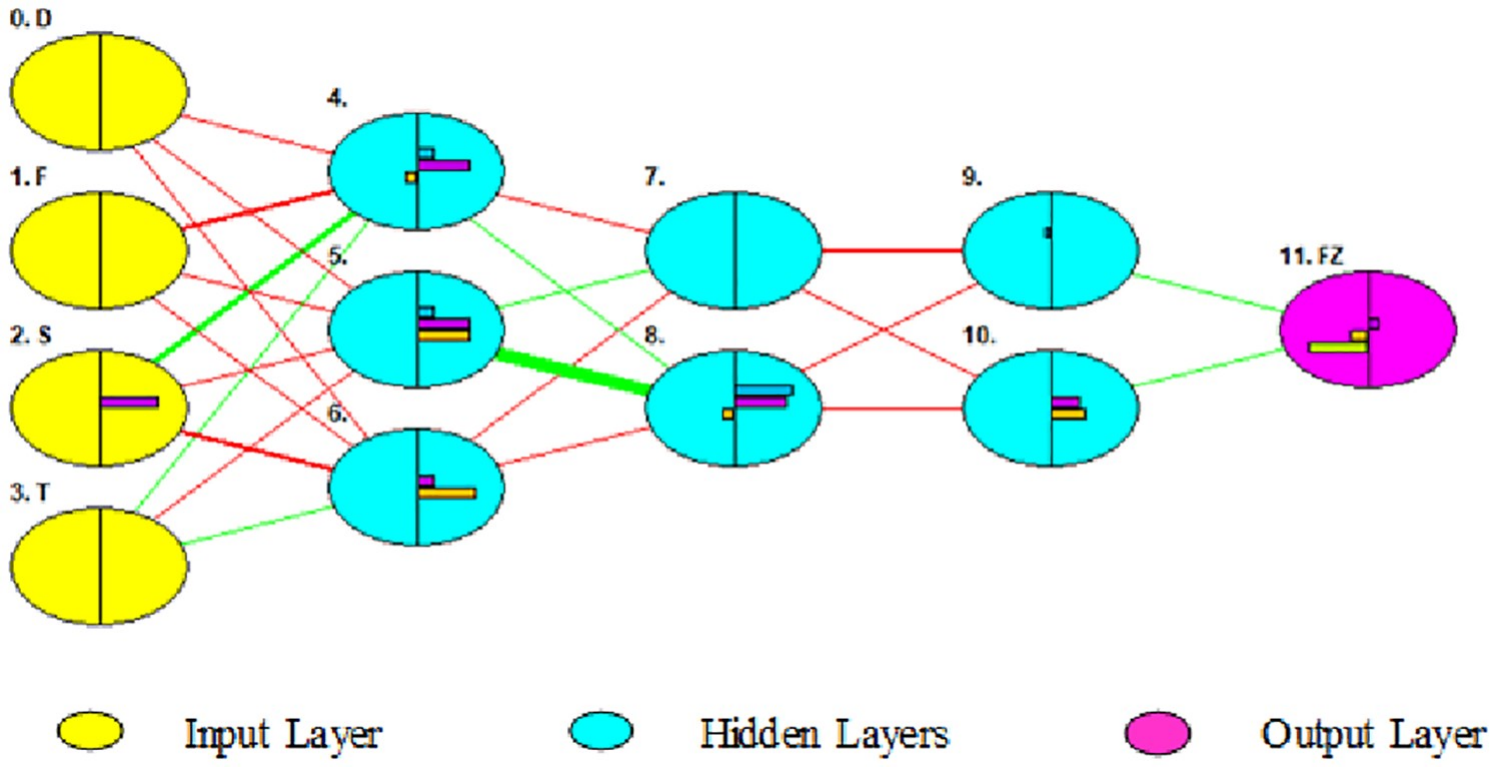
Neural network model for Fz.

The ANN computational model involves three layers, output, hidden, and input layers. Each layer contains neurons and each neuron is related to all the neurons in the next layer. Fig 3 shows the layers in a model of the forming force (Fz).

None of the processes are executed in the input layer, and the input for the neuron is obtained from the actual setting. The input vector is the weight of a neuron multiplied by the strength; the result obtained helps create the product. The output from the last neuron can be interconnected to the input of the next neurons or can be directly interconnected with the environment. The output comprises an activation function and a summation function. The activation function takes the weight of a neuron as an input and produces its activation as an output. The calculation of the net input from the processing neurons is the summation function. Using the ANN, the nonlinear relationships between the output and input owing to the contained activation function of the nonlinear and linear algebraic equations can be stored. After the weight is altered by the activation function, the neurons that have moved to other neurons make up the next layer. The output of the activation function accepts the results, and then presents them to either the external network or to the neurons in the next layer. The network output is compared with the target having the applied input, and the difference between them is then considered an error. Moreover, algorithms of different networks are applied to decrease the error [33].

## Result and discussions

### Statistical analysis

An analysis of variance (ANOVA) was used to estimate the effects of all factors and their interaction on Fz. As a standard practice in ANOVA, terms with a p-value < α = 0.05 are considered significant. The ANOVA results, presented in Table 5, indicate that the factors d, s, and t; the two-way interactions d*f, d*s, and d*t; and the three-way interaction between d, s, and t have a significant effect on Fz. The value of the adjusted R-squared value shows that the model can explain 91% of the variations in the data, and that 9% of the variations originate from unknown nuisance factors.

**Table 5:** ANOVA results for Fz.

Source	DF	Adj SS	Adj MS	F-Value	P-Value
Model	9	6002399	666933	25.34	0
Linear	4	4071606	1017901	38.67	0
d	1	1083491	1083491	41.16	0
f	1	1050	1050	0.04	0.844
s	1	356930	356930	13.56	0.001
t	1	2630134	2630134	99.92	0
2-Way Interactions	4	1765076	441269	16.76	0
d*f	1	148506	148506	5.64	0.027
d*s	1	406858	406858	15.46	0.001
d*t	1	1197730	1197730	45.5	0
s*t	1	11982	11982	0.46	0.507
3-Way Interactions	1	165717	165717	6.3	0.02
d*s*t	1	165717	165717	6.3	0.02
Error	22	579085	26322		
Lack-of-Fit	6	218117	36353	1.61	0.208
Pure Error	16	360968	22561		
Total	31	6581484			
Model Summary	S 162.241 R-sq 91.20% R-sq(adj) 87.60%				

### ANFIS results

The ANFIS model was developed as a function of SPIF for the forming force using training and testing data. The ANFIS tool that already exists in MATLAB was applied, which tests the relationship of the process parameters used to execute the perfect training and maximizes the prediction model accuracy for the selected responses (forming force). To obtain the results, the ANFIS algorithm was designed using the initial parameters. Table 6 lists the parameters used to help build the ANFIS model.

**Table 6:** Initial parameters for the construction of the ANFIS.

Responses	Forming force		
	Fx	Fy	Fz
Training method	hybrid	hybrid	hybrid
Membership function for inputs	gaussmf	trimf	psigmf
Number of membership function	3 2 3 1	2 2 2 2	3 3 3 3
Output function	constant	constant	constant
Number of epochs	100	100	100

**Fig 4:**
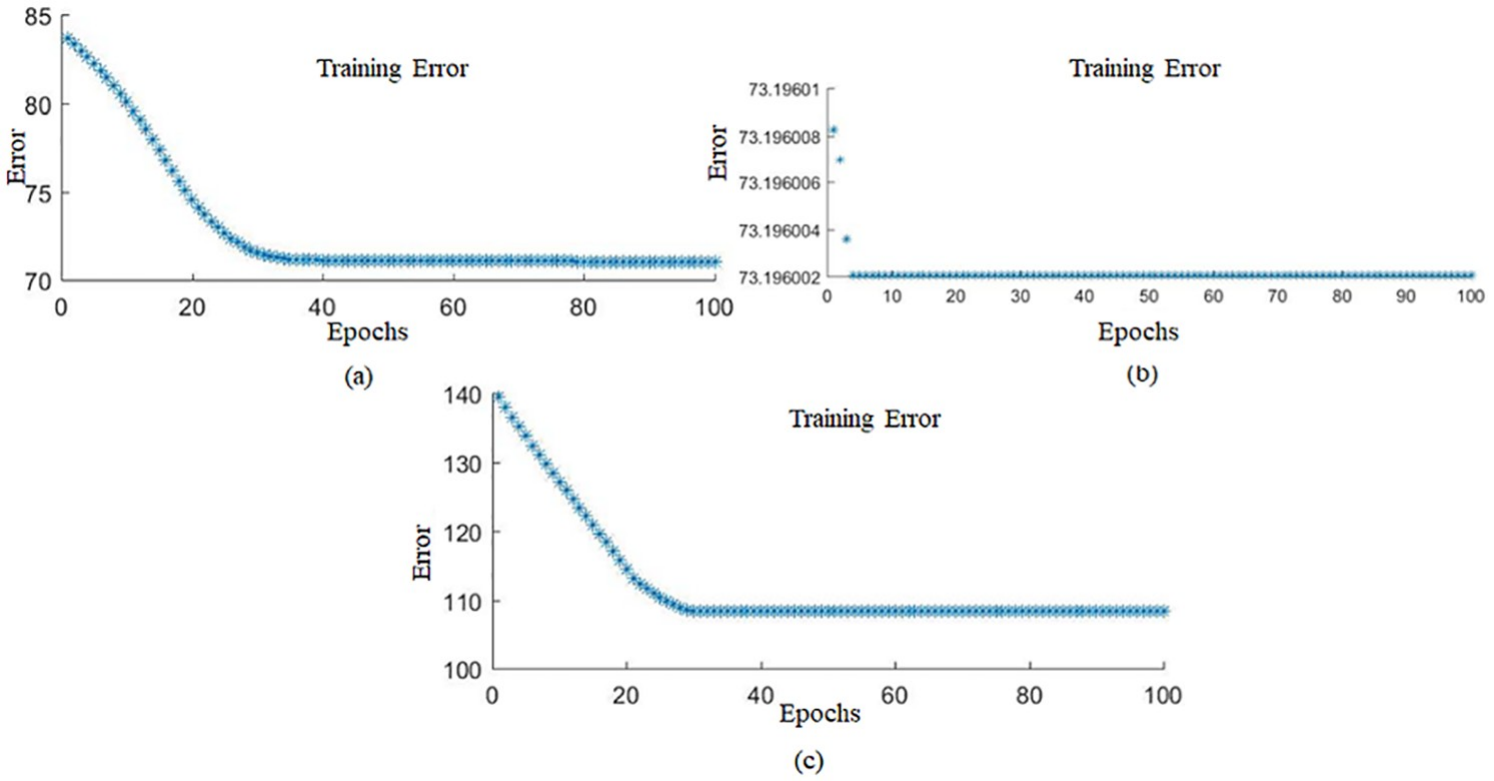
ANFIS training curve for forming force: (a) Fx, (b) Fy, and (c) Fz.

The training process was applied using 100 epochs for the forming force on three axes (Fx, Fy, and Fz). A training curve was obtained after the training process was complete, as shown in Fig 4. The figure shows the relationship between the number of epochs and the errors in the responses.

An analysis of the curves shows that, after 35 epochs, the errors become steady, as shown in Fig 4(a). This occurs because the developed model was trained using limited experimental data. To obtain the initial predicted values of the outputs, such as the forming force, a set of fuzzy inference parameters (FIPs) were selected during the training process. The measured values were compared with the predicted value of the forming force obtained from the developed ANFIS model. The performance of this model was measured based on the difference between the measured and predicted values.

**Fig 5:**
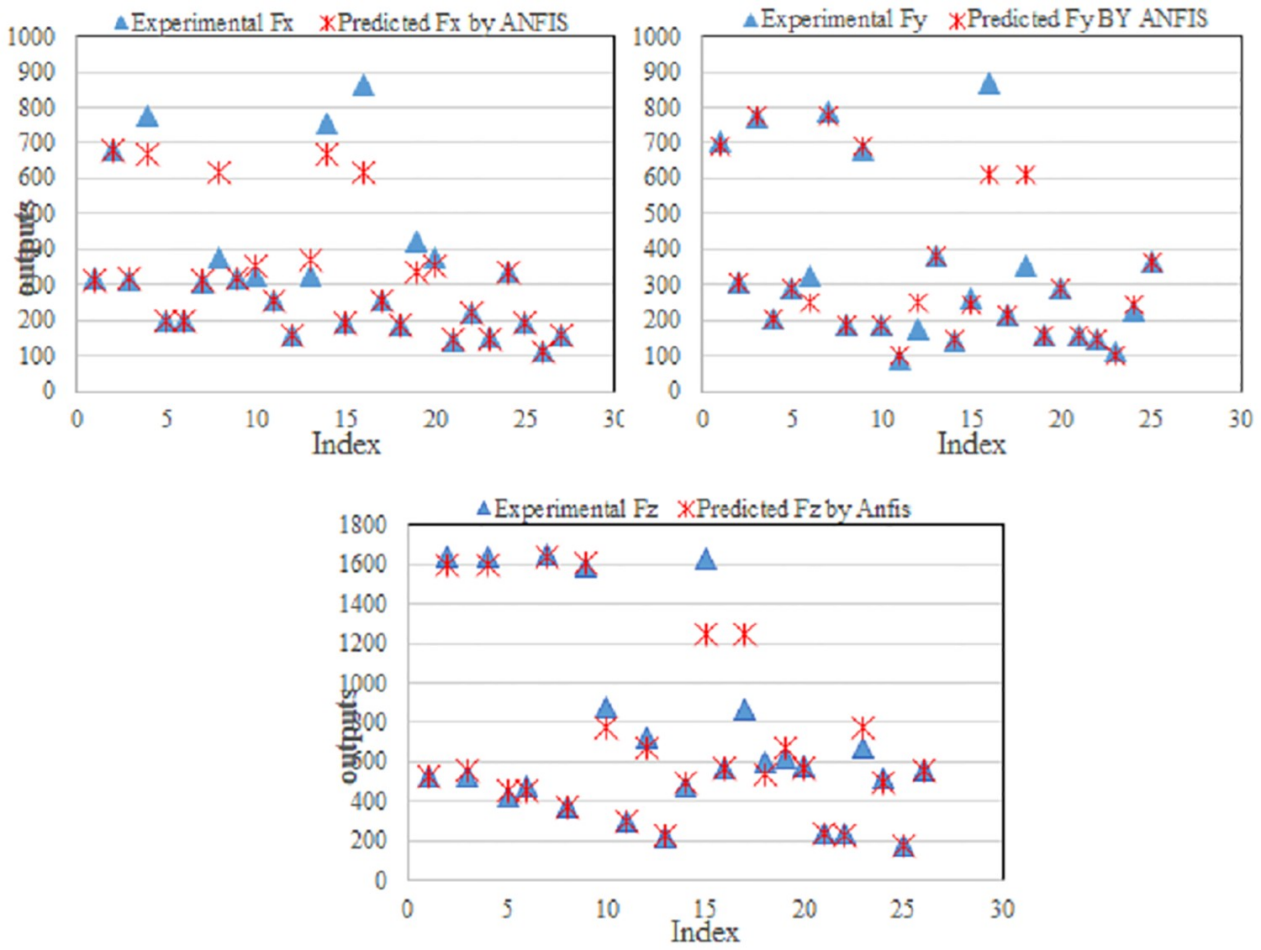
Comparison between the measured and predicted forming forces using the ANFIS training data.

**Fig 6:**
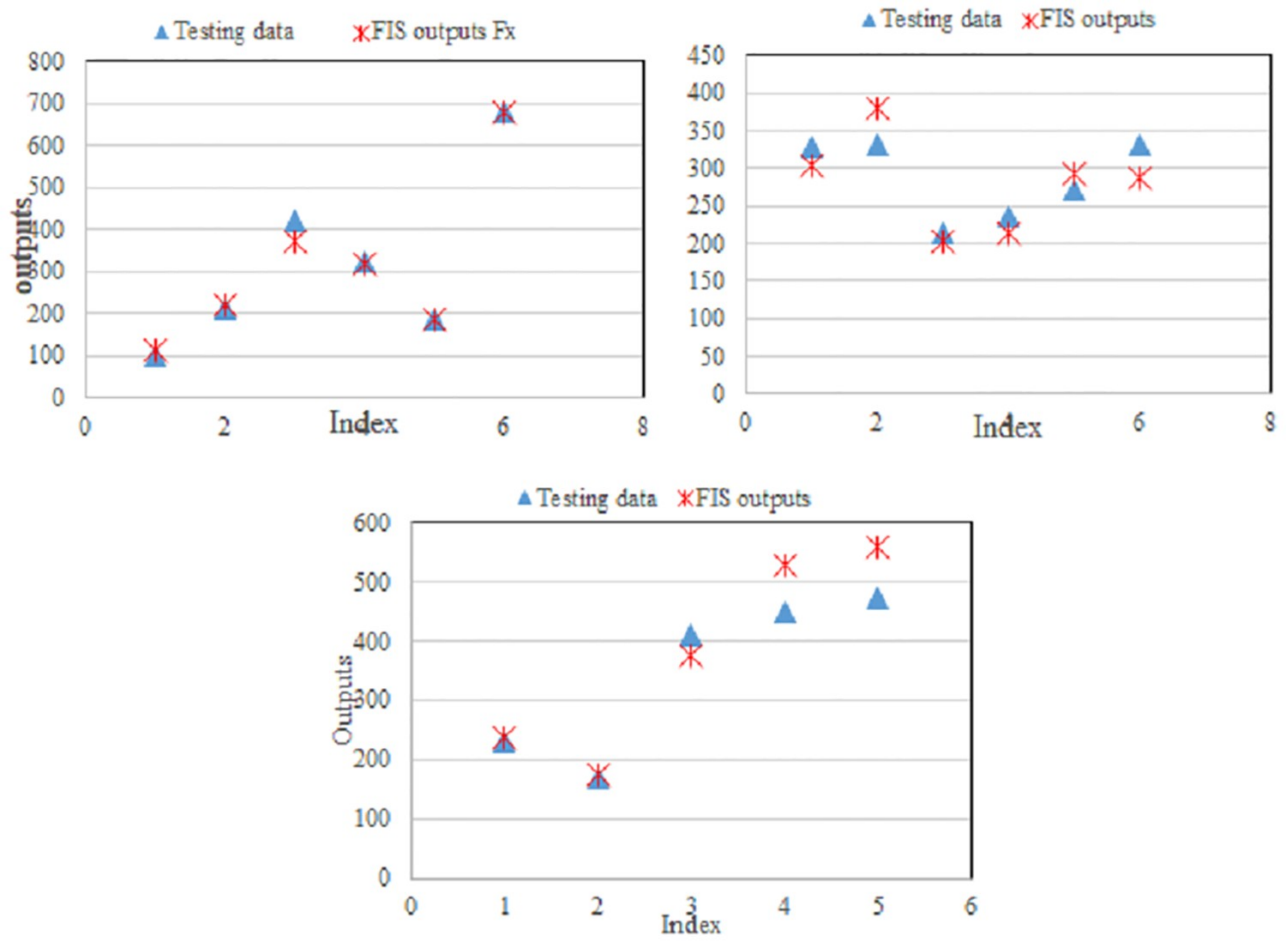
Comparison between the measured and predicted forming force using the ANFIS test data.

During the training process, FIPs were repeated multiple times until the errors were minimized. Different ANFIS parameters were used as the training parameters to validate the accuracy of the prediction model. Table 7 shows the different ANFIS architectures for a predictive model of the forming forces obtained for different input membership shapes, numbers of membership functions, and types of output (linear or constant). For instance, from Table 7, the trimf function was chosen to train the ANFIS because it achieved the lowest testing error of 31.4218. In addition, Figs 5 and 6 show a comparison between the measured and predicted forming forces for the training and testing data.

**Table 7:** Different ANFIS architectures for forming force.

Responses	NO. MF	Type of MF	Output function	Errors RMSE	
				Training error	Test error
Forming force (Fx)	3 3 3 3	trimf	constant	73.196	31.4218
			linear	73.196	31.4166
	2 2 2 2	Trapmf	constant	73.4195	33.2606
	3 3 3 3		constant	73.196	31.4218
	3 3 3 3	psigmf	constant	73.196	31.4309
Forming force (Fy)	2 2 2 2	trimf	constant	108.9255	103.2906
	2 2 2 2		linear	108.3141	104.6017
	3 3 3 3	trapmf	constant	108.3141	104.5998
	2 2 2 2		constant	108.3236	104.2812
	2 2 2 2	psigmf	constant	108.3145	104.5399
	3 3 3 3		constant	108.3141	104.5998
Forming force (Fz)	3 3 3 3	psigmf	Constant	70.7654	32.4088
	3 3 2 1		linear	96.5383	105.72
	2 2 2 2	gaussmf	constant	70.2728	31.9748
	3 3 3 3		constant	71.018	37.5940
	3 3 3 3	trapmf	constant	71.0169	31.8061
	3 2 3 1	gaussmf	constant	172.7283	131.9702

### Artificial neural network results

The results of the developed ANN model are used to predict the forming force based on the input process parameters in single-point incremental sheet metal forming. The numbers of training and testing samples are 28 and 6, respectively.

**Fig 7:**
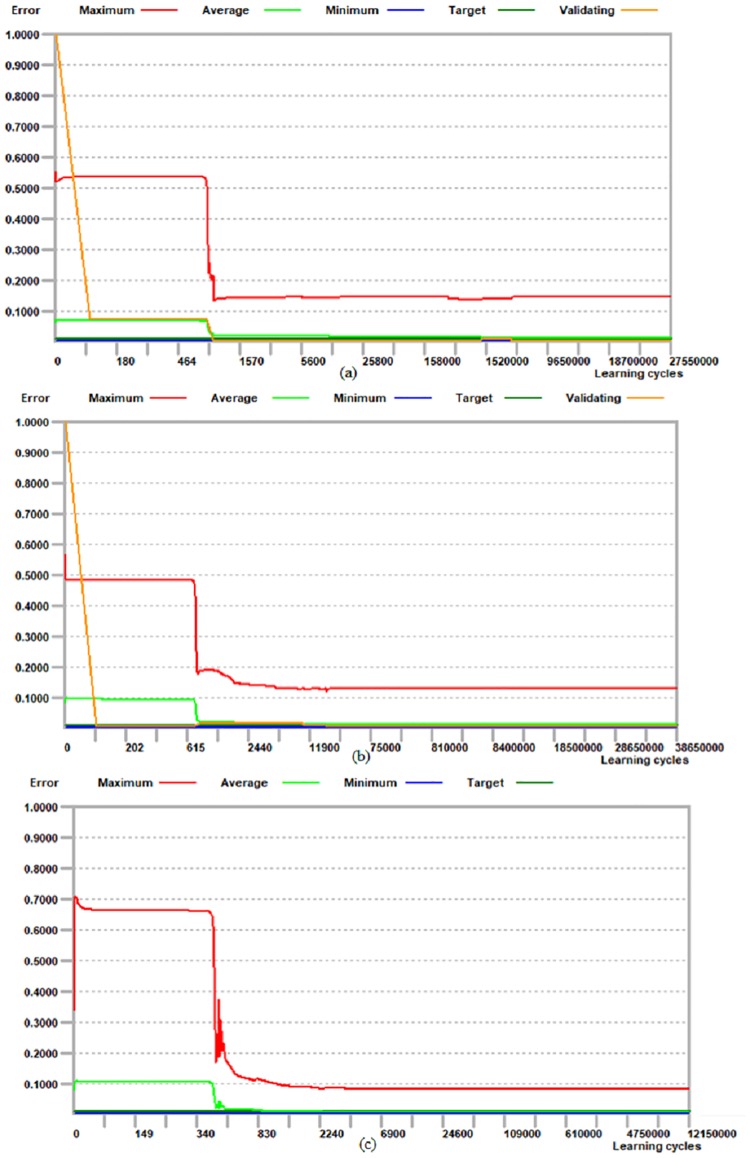
ANN training curve for forming force.

Several training experiments were carried out to identify the optimal network structure and best training parameters of the neural networks, producing minimum errors during the training phase. Similarly, several training experiments with different numbers of hidden neurons, learning rates (0.60), and momentum values (0.80) were checked, as shown in Fig 7. The graph of the learning progress shows the maximum, average, and minimum training errors. The average validation error is 0.00138, which was obtained for a maximum of 38,650,000 learning cycles. The correlation coefficient (R value) can be used to gauge the performance of the established network. The R value is between the measured value and the predicted value for the testing (6) and training data (28). The measurement of the closeness of the dissimilarity in the output clarified by the target is known as the R value, which lies between 1 and 0. When the R value equals 1, the optimal correlation is observed between the output and target values for the forming force on the three axes. The R value obtained between the predicted values and the measured data is 0.981, which indicates a good correlation.

### Regression model

A regression analysis helps in the development of a mathematical equation to characterize the relationship between two or more input variables and the response outputs. In this study, mathematical models are also developed using a regression analysis to fit the measured data for the three selected responses. Using Minitab software, regressions models for the forming force were developed, and a full quadratic model was initially selected for all responses. Later, the insignificant terms were removed based on their p-values and accuracy. The following equations can be used to predict the forming force as a function of significant factors:

$$F_{x}(N)=-814+70.0d+0.981f+2009\;s-254.2\;t-0.0749d*f-149.4\;d*s+30.86\;d*t-1.815\;f*s+0.1282\;d*f*s,$$ (6)

$$F_{y}(N)=-441+16.5d+0.605\;f+1735\;s+259\;t+0.0250\;d*f-44.8\;d*s+32.93\;d*t-32.93\;f*s-0.663\;f*t-782\;s*t+1.015\;f*s*t,$$ (7)

$$F_{z}(N)=2650+182.0\;d-\;0.795\;f+-\;1428\;s-1767\;t+\;0.0545\;d*f+\;82.5\;d*s+163.7\;d*t+\;1572\;s*t-\;115.1\;d*s*t,$$ (8)

## Comparison of ANFIS with ANN and regressions

**Fig 8:**
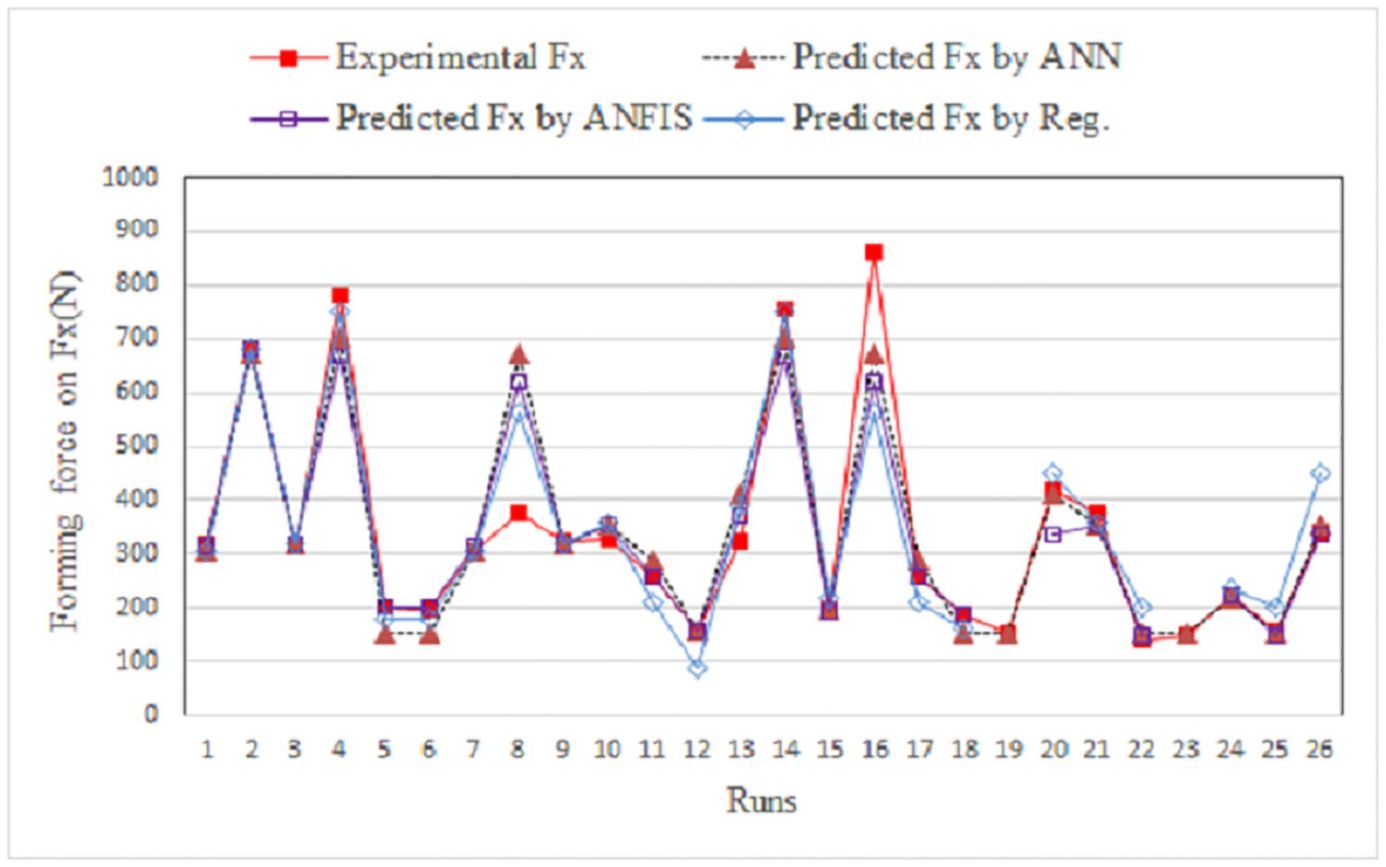
Comparison between experimental and predicted Fx for training data.

**Fig 9:**
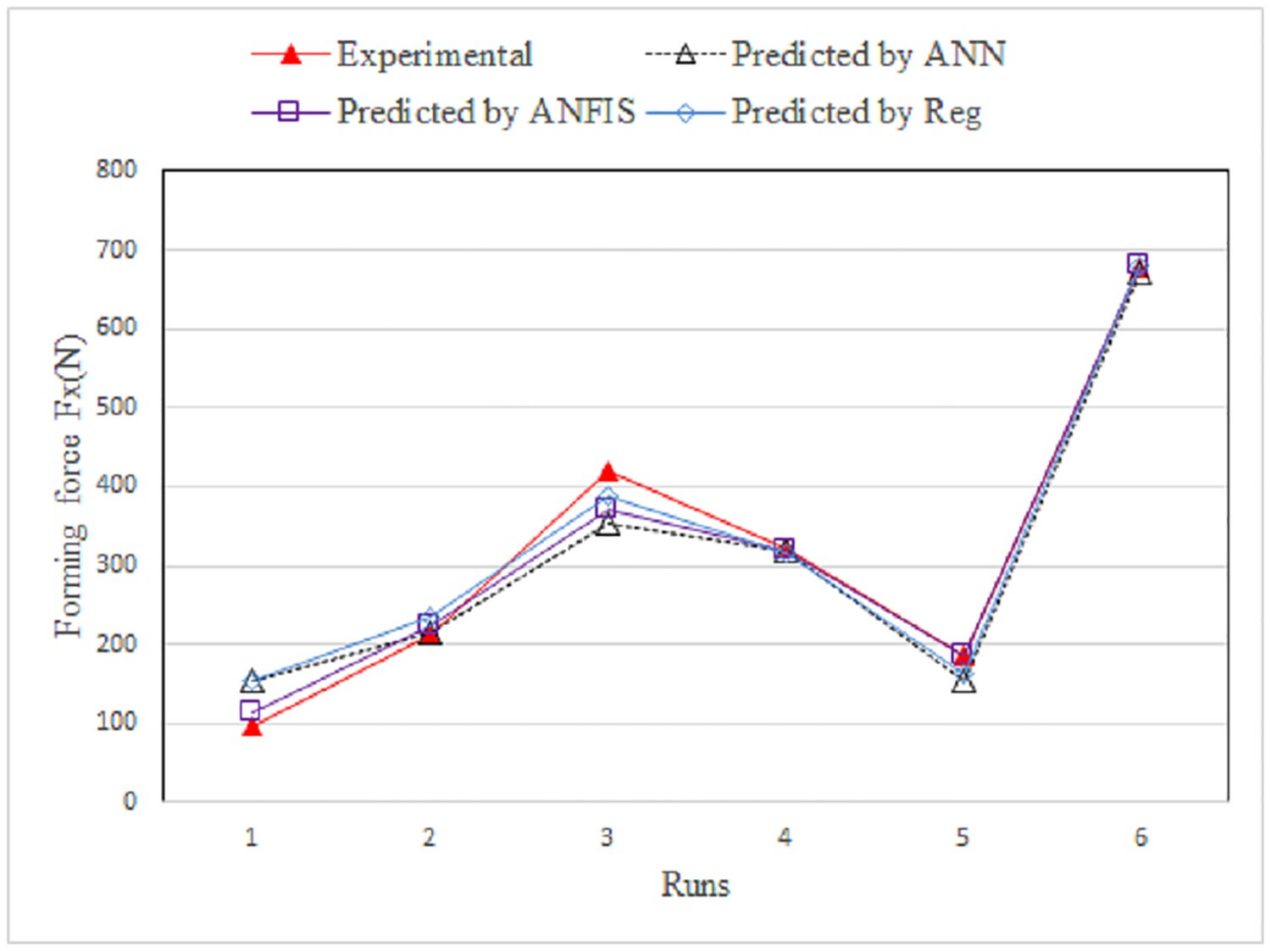
Comparison between measured and predicted Fx for testing data.

To assess the ability of the developed ANFIS model relative to that of a neural network and regression analysis, an ANN model and a regression algorithm were developed using the same input variables. Table 4 summarizes the results. For the forming force model along the x-axis, Fig 8 shows a comparison between the measured and predicted values obtained using the ANFIS, ANN, and regressions models for the training data. Fig 9 shows the same for the testing data.

**Fig 10:**
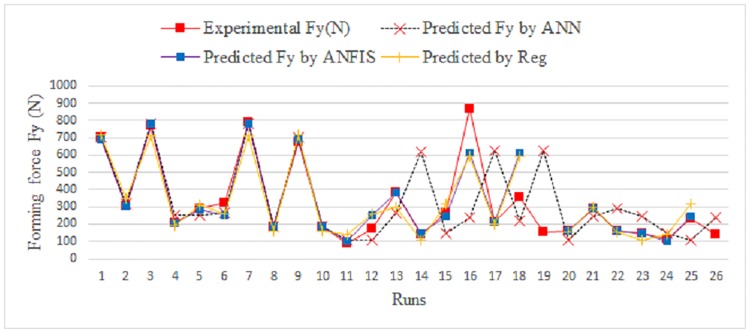
Comparison between measured and predicted Fy for training data.

**Fig 11:**
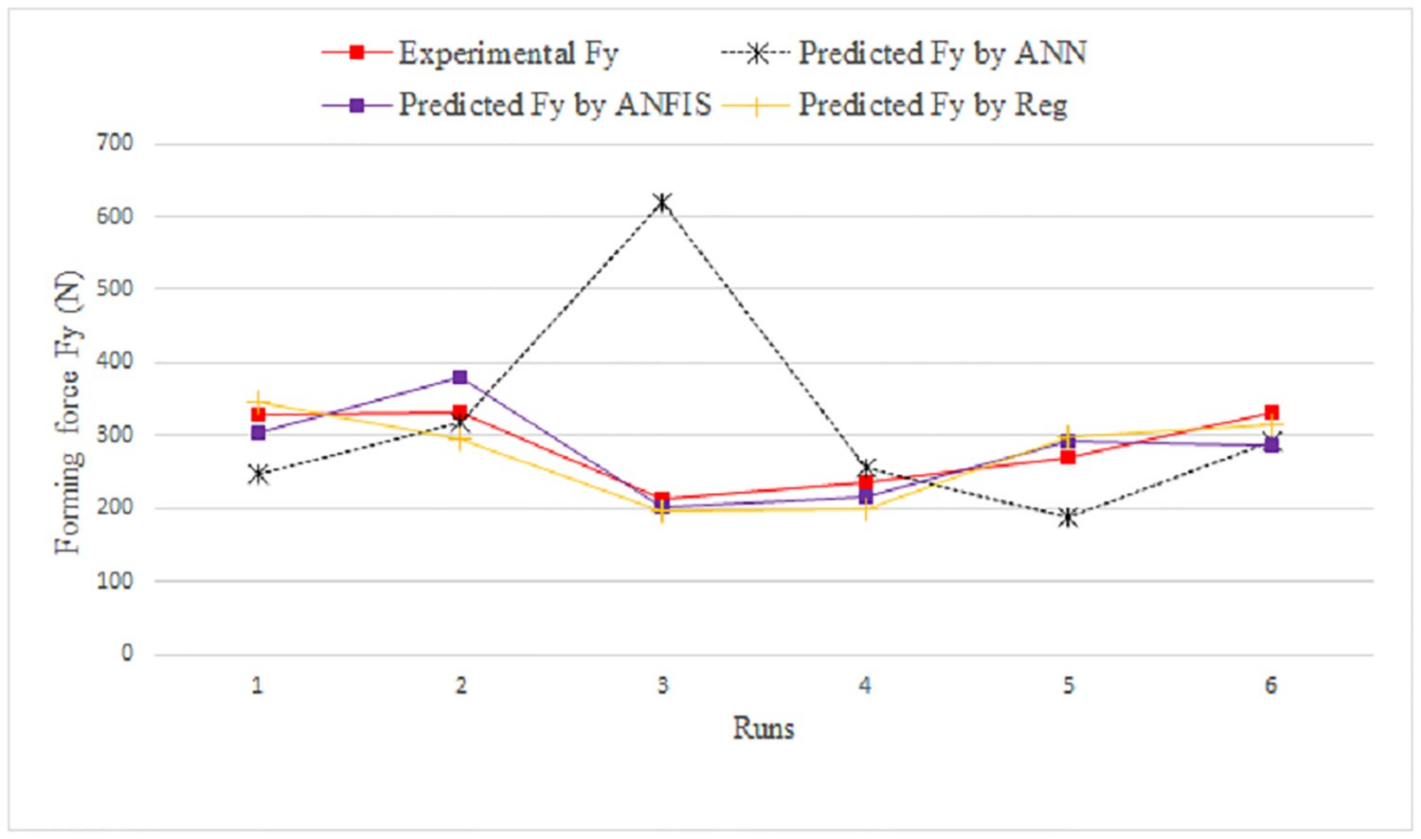
Comparison between measured and predicted Fy for testing data.

Fig 10 shows a comparison between the measured and predicted values obtained using ANFIS and the regression model for the training data. Fig 11 shows the same for the testing data.

**Fig 12:**
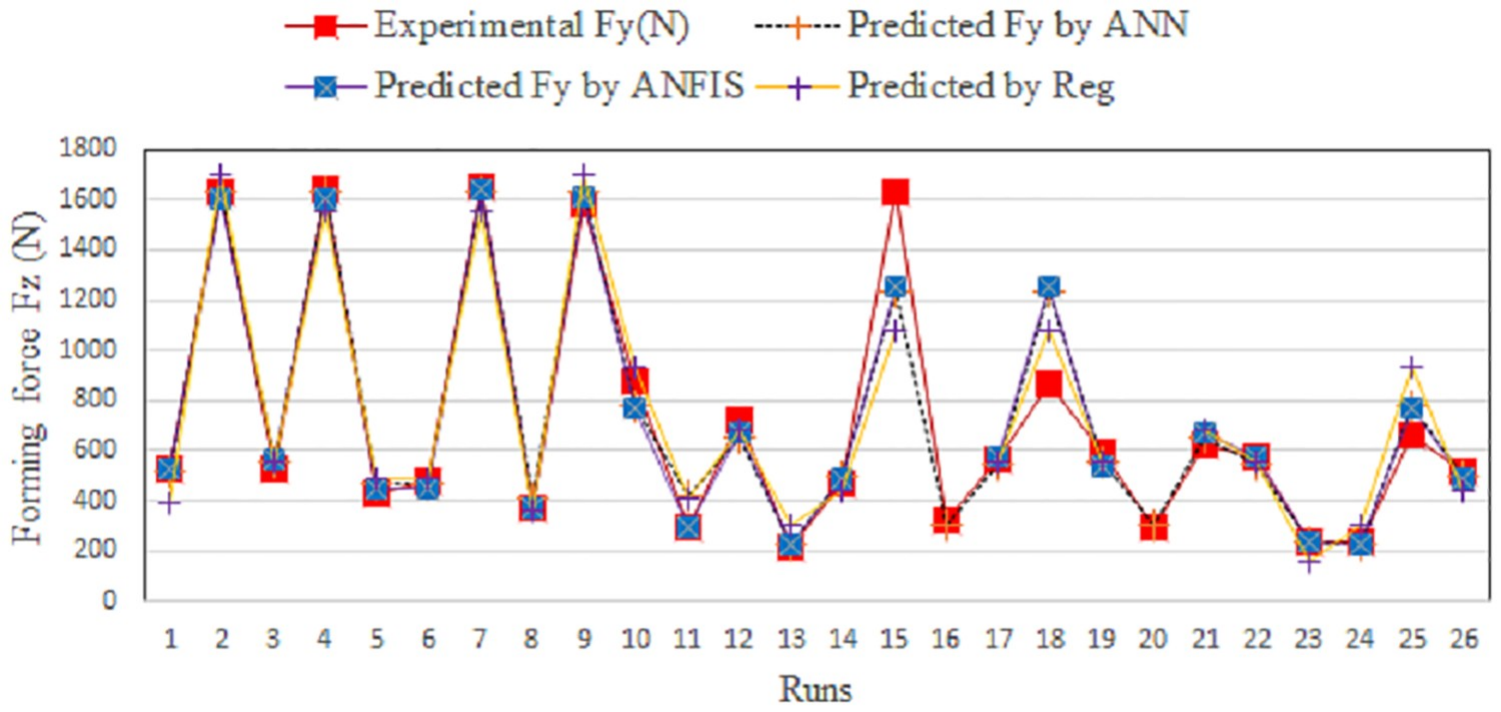
Comparison between measured and predicted Fz for training data.

**Fig 13:**
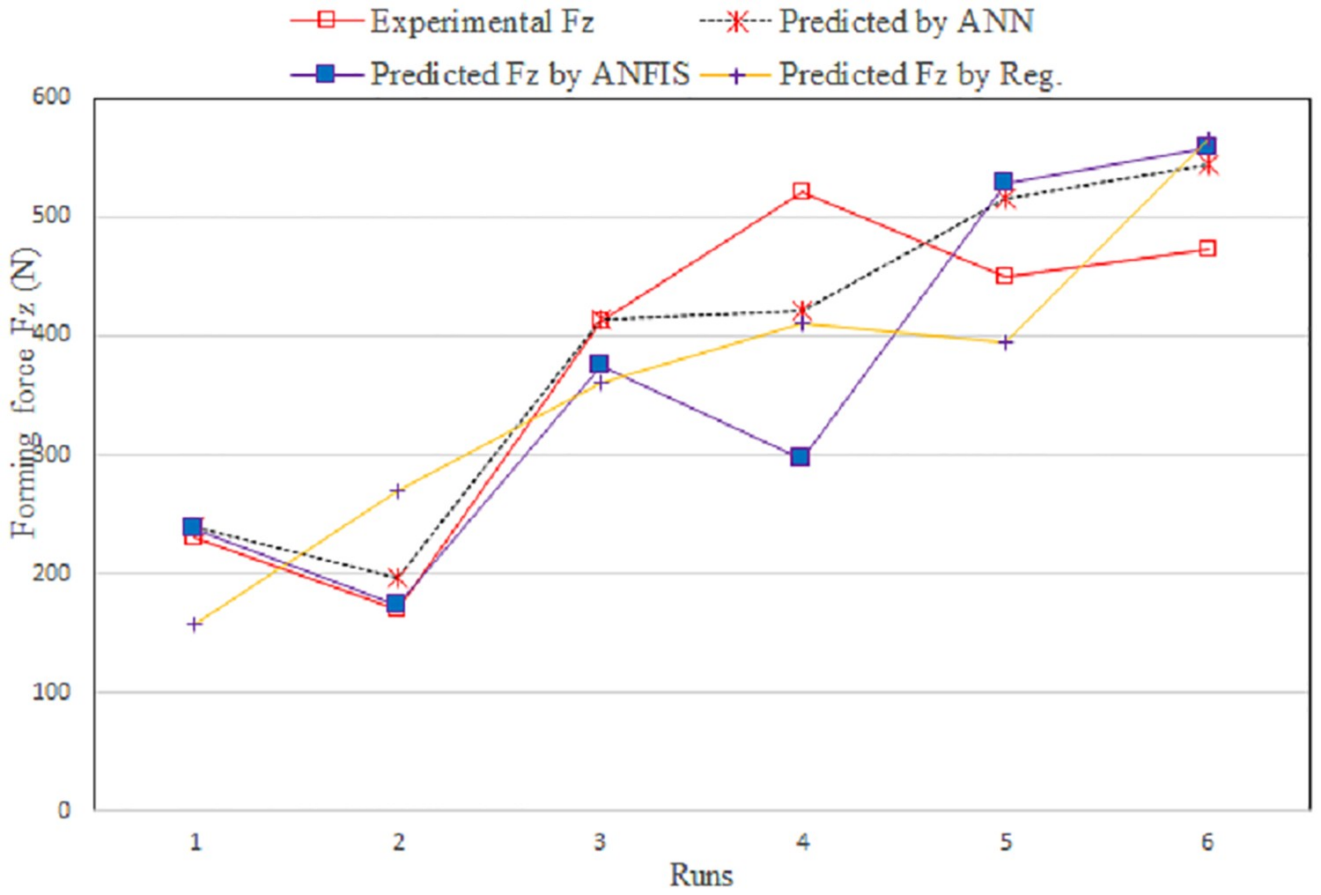
Comparison between experimental and predicted Fz for testing data.

Fig 12 shows a comparison between the measured and predicted values obtained using ANFIS and the regression model for the training data. Fig 13 shows the same for the testing data.

The results obtained using the ANFIS prediction are very close to the measured values. Moreover, the absolute mean percentage errors were calculated for each of the developed models. Tables 8 and 9 present a comparison of the performance between the ANFIS, ANN, and regression models based on the mean absolute percentage errors (MAPE) for the training and testing data.

**Table 8:** Comparison of the developed models based on the mean absolute percentage errors for training data.

Outputs	ANFIS model	ANN model	Reg. model
	MAPE	MAPE	MAPE
Forming force Fx	7.25	12.04	18.08
Forming force Fy	8.25	55.73	19.55
Forming force Fz	6.42	8.98	16.27

**Table 9:** Comparison of the developed models based on the mean absolute percentage errors for testing data.

Outputs	ANFIS model	ANN model	Reg. model
	MAPE	MAPE	MAPE
Forming force Fx	5.85	16.14	15.37
Forming force Fy	9.61	44.77	9.42
Forming force Fz	15.44	11.59	26.05

Based on the performances of the ANFIS and ANN models in terms of the average absolute percentage error for the training and testing data, it was observed that the ANFIS model outperforms the ANN and regression models, while retaining their full potential.

## Conclusions

This paper proposed ANFIS and ANN models to predict the forming force in the context of sheet metal forming, particularly SPIF. In addition, the influences of the tool diameter, feed rate, sheet thickness, and step size on the main forming force were investigated. Considering the ANOVA for the forming force (Fz), it was concluded that the significant factors are the tool diameter, step size, and sheet thickness. The results of the ANFIS and ANN models were compared with both the experimental data and those predicted using a regression model. The comparison showed that the ANFIS model can accurately predict the forming force for both training and testing data; in addition, the ANFIS model exhibited a better prediction performance for the selected responses. Moreover, the results showed that the ANFIS model can predict the forming force along the three axes for the training data with a MAPE of 7.25%, 6.42%, and 8.98%, respectively, and for the testing data with a MAPE of 5.85%, 9.61%, and 15.44%, respectively. It can therefore be concluded that the developed model using the ANFIS approach can be effectively used to measure the forming force during ISF and provide more reliable results than the ANN and regression models.

## Abbreviations

ANFIS: Adaptive neuro-fuzzy inference system
ANN: Artificial neural network
Dsigmf: Difference of two fuzzy sigmoid membership functions
Fx: forming force on x-axis
Fy: forming force on y-axis
Fz: forming force on z-axis
Gaussmf: Gaussian membership function
Gbellmf: Generalized bell membership function
MAPE: Mean absolute percentage error
Psigmf: Product of two sigmoidal functions
Trapmf: Grapezoidal membership function
Trimf: Triangular membership functions
